# Enlarged airspaces in the distal lung in adolescents born very preterm as measured by aerosol

**DOI:** 10.1136/bmjresp-2024-002666

**Published:** 2024-12-22

**Authors:** Hugo Öhrneman, Frida Lindström, Cecilia Hagman, Madeleine Petersson Sjögren, Jenny Rissler, Per Wollmer, Ellen Tufvesson, Jakob Löndahl

**Affiliations:** 1 Department of Design Sciences, Lund University, Lund, Sweden; 2 Department of Clinical Sciences, Lund University, Lund, Sweden; 3 Department of Clinical Sciences Lund Paediatrics, Skåne University Hospital Lund, Lund, Sweden; 4 Clinical Physiology, Skånes universitetssjukhus Malmö, Malmo, Sweden

**Keywords:** Respiratory Measurement, Emphysema, Paediatric Lung Disaese

## Abstract

**Rationale:**

Preterm infants diagnosed with bronchopulmonary dysplasia (BPD) are thought to have fewer and larger alveoli than their term peers, but it is unclear to what degree this persists later in life.

**Objectives:**

To investigate to what degree the distal airspaces are enlarged in adolescents born preterm and to evaluate the new Airspace Dimension Assessment (AiDA) method in investigating this group.

**Methods:**

We investigated 41 adolescents between 15 and 17 years of age, of whom 25 were born very preterm (a gestational age <31 weeks, with a mean of 26 weeks) and 16 were term-born controls. Of the preterms, 17 were diagnosed with BPD. The AiDA method was used to measure the average distal airspace radius (r_AiDA_) in the lungs. In addition, lung function was evaluated by spirometry, impulse oscillometry and diffusing capacity of carbon monoxide (D_LCO_).

**Measurements and main results:**

We observed a mean r_AiDA_ of 295±53 µm for the preterm group compared with 231±12 µm for the control group (p<0.0001). The adolescents diagnosed with BPD had a mean r_AiDA_ of 313±54 µm. There was a strong negative correlation between gestational age and distal airspace radius (p<0.0001). The BPD group had a decreased FEV_1_ (forced expiratory volume in 1 s, z-score: −1.28±1.37, p=0.012) and D_LCO_ (z-score: −0.92±1.01, p=0.013) compared with the controls, but all other lung function variables showed normal values.

**Conclusions:**

Our results suggest that the enlarged airspaces seen in preterm infants likely remain in adolescence. Distal airspace radius as measured by AiDA was the lung function variable that showed the most significant difference between preterm and term-born adolescents.

**WHAT IS ALREADY KNOWN ON THIS TOPIC**

- Preterm birth is known to cause respiratory complications during infancy, and many preterm infants are diagnosed with bronchopulmonary dysplasia (BPD). To what degree this persists throughout life is still unclear.

**WHAT THIS STUDY ADDS**

- This study shows that preterm-born adolescents have significantly larger distal airspaces than their term-born peers. Those with BPD had the largest airspaces and the airspace radius was significantly correlated with gestational age. This was observed with the new method Airspace Dimension Assessment (AiDA), which shows differences between groups that spirometry, impulse oscillometry and diffusing capacity of carbon monoxide did not capture to the same degree.

**HOW THIS STUDY MIGHT AFFECT RESEARCH, PRACTICE OR POLICY**

- Our findings indicate that individuals born prematurely have underdeveloped distal airspaces, a condition that seems to persist into adolescence. AiDA shows promise in investigating this patient group without the need for CT or MRI which are more costly and may entail ionising radiation.

## Introduction

 As a result of improvements in neonatal care, a higher number of prematurely born children survive into adulthood. In high-income countries, about 1% of all births are estimated to be very preterm (ie, a gestational age of less than 32 weeks) and the incidence is projected to increase.^1^ Therefore, it is important to understand the health outcomes related to preterm birth. Impaired lung function during infancy is a common consequence of preterm birth and the continued development of the lungs is not fully understood.^2^

Many children who are born prematurely are diagnosed with bronchopulmonary dysplasia (BPD), first described by Northway *et al*.^3^ The diagnosis is based on a need for additional oxygen after preterm birth. BPD is a diagnosis that has changed over time, which is reflected in the altered definitions of the diagnosis, going from ‘old’ to ‘new’ BPD in 2001,^4^ to a refinement in 2018 taking more modes of ventilation into consideration.^5^

Most research on BPD has focused on effects during infancy, but the diagnosis is also associated with an increased prevalence of respiratory issues throughout life.^6 7^ Children born preterm without the BPD diagnosis also often exhibit decreased lung function,^8^,^12^ suggesting that gestational age alone could play an important role in long-term respiratory health.

Autopsies on infants have shown that those diagnosed with BPD have fewer and larger alveoli as a consequence of an arrest in alveolar development.^13^ These autopsies were taken from very severe cases of BPD, and there is currently a need to study this non-invasively in survivors of preterm birth. For infants born very or extremely prematurely, the arrest in development occurs during a period when the lungs are still in the saccular stage of development, prior to secondary septation of the alveoli, which can lead to underdeveloped and larger alveoli for preterm children. In addition to structural underdevelopment, there is considerable growth of the vascular system in the lung between 22 and 32 weeks of gestation. Interruption of angiogenesis in preterm born is likely to affect the gas exchange in the lung, as well as impairing alveolarisation, as blood vessels in the lung have been suggested to promote alveolar growth and aid in the maintenance of alveolar structures throughout postnatal life.^14^ It remains unclear whether those born prematurely can regain some lung function later in life and, if so, whether this is due to structural changes in the distal lung or due to the body compensating in other ways.

It is difficult to measure structural changes in the distal lung without advanced and costly methods such as CT or MRI. CT also has the added drawback of radiation exposure. In a review of 19 CT studies, Van Mastrigt *et al* reported structural abnormalities in over 85% of patients with BPD.^15^ One of the reviewed studies by Ronkainen *et al* found that 81% of BPD subjects, aged 8–16, had abnormalities on CT.^16^ Hypoattenuation and opacities were the most common findings, which have been related to abnormalities in the peripheral lung.^15^ Less costly methods, such as spirometry or diffusing capacity of carbon monoxide (D_LCO_), are insufficient to study the structure of the distal lung since they are affected by the conductive airways or by factors affecting the gas exchange, such as vascularisation.^17^ Recently, the Airspace Dimension Assessment with inhaled aerosol (AiDA) technique has been developed to measure the distal airspaces in a simple, non-invasive manner and has previously been able to separate healthy controls from patients with emphysema.^18^,^20^ AiDA exploits the tendency of inhaled particles with a 50 nm diameter to deposit distally to generation 15 in the respiratory tree. The deposition time of a particle depends on its residence time and the airspace size. By controlling the residence time, one can estimate the average size of the airspaces.

The objective of this work was to investigate lung function in adolescents born preterm, with and without BPD, using AiDA and standard lung function tests, including spirometry, impulse oscillometry (IOS) and D_LCO_.

## Methods

### Participants

The preterm participants were recruited from two cohorts born 2004–2007 at gestational age <27 weeks (the Swedish national EXPRESS cohort^21^) and <31 weeks.^22^ Term-born controls were enrolled from the EXPRESS cohort or recruited locally.

Lung function for the cohort had previously been assessed at the age of 12.^12^ Participants who were able to perform lung function tests at 12 years of age and non-asthmatic term-born controls who at that time had a forced expiratory volume in 1 s (FEV_1_) above 95% of predicted, were eligible for inclusion in the present study. The limit of 95% of the predicted value was chosen to eliminate subjects with an undiagnosed airway obstruction.

Due to living out of reach or lack of a valid address, 14 preterm and 10 term-born adolescents were excluded. Out of 45 preterm born who were asked to participate, 20 declined participation, while 25 adolescents (8 without and 17 with a diagnosis of BPD) were included. In addition, 22 term-born controls were asked to participate, of which 12 declined participation and 1 was excluded due to having developed asthma. Furthermore, eight term-born controls were recruited locally, of which one was excluded due to having an FEV_1_ below the lower limit of normal. In total, 16 term-born adolescents were included.

BPD was in this study defined as having been treated with extra oxygen at 36 weeks postmenstrual age. Birth weight deviation and small for gestational age was calculated from equations determined by Marsál *et al*.^23^ Among the preterm-born participants with available data, all had received continuous positive airway pressure (CPAP) as infants and 18 out of 25 had received mechanical ventilation. The duration of CPAP treatment was unavailable for six subjects.

### Study design

At the age of 15–7 years, participants performed AiDA, spirometry, IOS and D_LCO_ measurements during the same visit. Height and weight at the time of measurement were compared against reference values determined by Wikland *et al*.^24^ For subjects’ characteristics see table 1.

**Table 1:** Participant characteristics

	Term-born controls (n=16)	Preterm without BPD (n=8)	Preterm with BPD (n=17)
At the time of the study visit (15–17 years old)			
Male/female (n)	7/9	3/5	10/7
Age (years)	16.4±0.6	16.2±0.4	16.6±0.6
Height (cm)	169±7	169±10	167±6
Height (z-score)	−0.56±0.71	−0.51±0.92	−1.05±1.08
Weight (kg)	58.5±7.3	60.2±11.4	60.2±11.4
Weight (z-score)	−0.44±0.68	−0.23±1.11	−0.33±1.51
Asthma (n)	0‡	1	5
Neonatal data			
Gestational age (weeks+days)	39 w+2 d±10 d†	27 w+4 d±13 d***	25 w+6 d±14 d***
Small for gestational age (n)	NA	2	3
Weight at birth (g)	3521±562‡	1081±348***	825±225***
Birth weight deviation (z-score)	0.18±1.20	−0.76±1.38	−0.85±1.23
Days with mechanical ventilation	NA	6±13	11±11
Days with CPAP	NA	20±13^†^	46±16§

Data are presented as number (n) or means and standard deviationsSD.

*p<0.017 compared tocompared with controls; **p**p<0.003 compared tocompared with controls; ***p***p<0.0003 compared tocompared with controls.

†dData missing from one person.

‡††dData missing from two persons.

§†††dData missing from six persons.

Birth weight deviationdeviation from expected birthweight at that gestational ageBPD, bronchopulmonary dysplasia; CPAP, continuous positive airway pressure; d, days; small for gestational agea birthweight at least 2 SDs below the mean for their gestational agew, weeks

### Patient and public involvement

Due to the exploratory nature of this research, it was not possible or practical to involve patients in the design, conduct, reporting or dissemination plans of this study.

### Airspace Dimension Assessment with inhaled aerosol

With the AiDA method distal airspaces are measured by analysis of inhaled and exhaled concentrations of 50 nm aerosol particles. The deposition fraction of this aerosol is calculated from inhaled and exhaled particle concentrations and relates directly to the size of the peripheral airspaces.^19 25^ The deposition fraction over several breath-holds is used to calculate an average airspace radius (r_AiDA_) and a zero-second recovery (R_0_), which is assumed to be determined by geometrical properties of the peripheral lung, such as airway heterogeneity. The breathing manoeuvre is similar to that used for single-breath D_LCO_, with the subject breathing into a mouthpiece while wearing a nose-clip. The subjects breathed particle-free air for approximately 30 s, after which air was exhaled to residual volume and then aerosol particles were inhaled to total lung capacity. Thereafter, the subjects held their breath for a few seconds, and then exhaled. The aerosol concentration was measured from exhaled air after the washout of dead space. The manoeuvre was repeated with breath-holds of 5, 7 and 10 s, with two successful replicates of each, for a total of six measurements.

**Figure 1:**
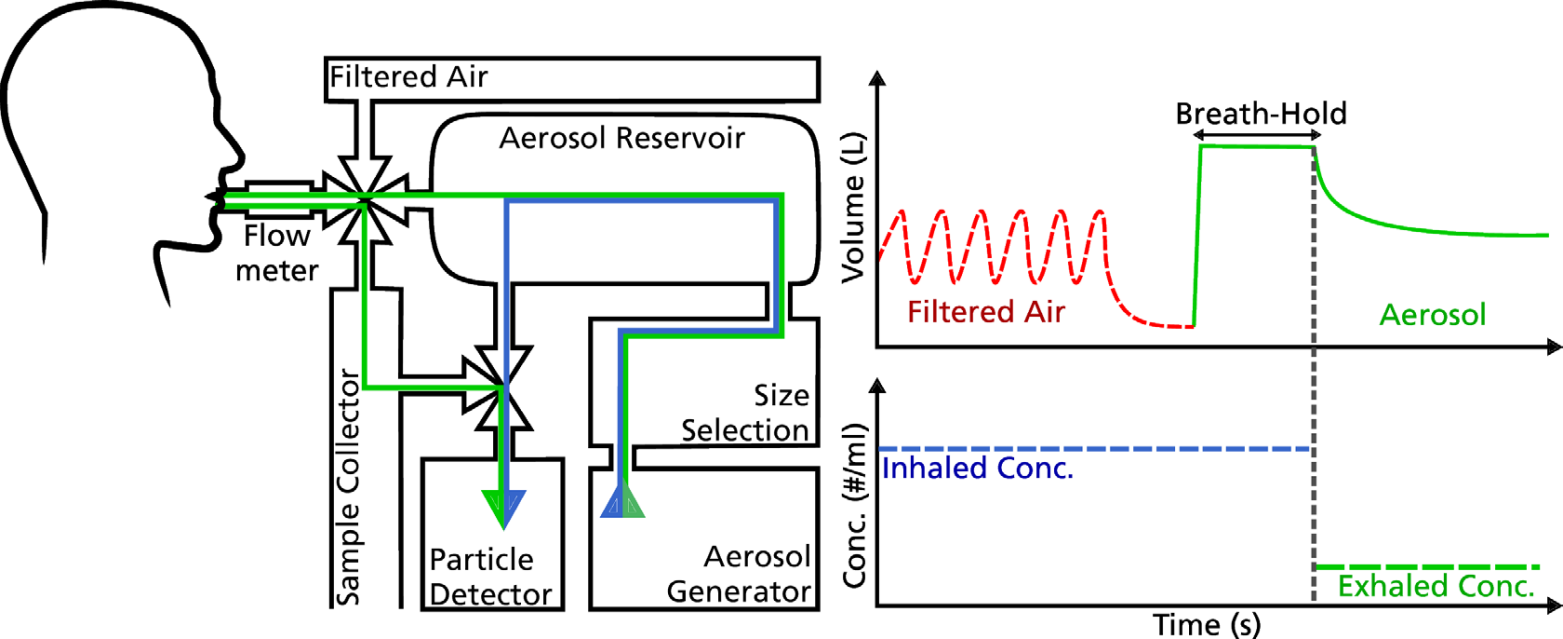
Schematic view of the Airspace Dimension Assessment system showing particle generation, size selection, inhalation system and concentration determination. Arrows show the path of the aerosol before (blue) and during inhalation (green). The diagram illustrates the breathing pattern and the measured particle concentration during one measurement.

A simplified diagram of the AiDA set-up can be seen in figure 1, which has been described in more detail elsewhere.^26^ Briefly, 50 nm polystyrene latex spheres were aerosolised using an electrospray aerosol generator (Model 3480, TSI, Shoreview, Minnesota, USA) and thereafter size was selected using a differential mobility analyser (Model 3071, TSI GmbH, Aachen, Germany). Inhaled and exhaled nanoparticle number concentrations were determined by a condensation particle counter (Model A20, Airmodus Oy, Helsinki, Finland). The distal airspace radius, r_AiDA_, was calculated from the nanoparticle diffusion coefficient for 50 nm particles and the half-life time of the inhaled particles, which is available from the slope of a least-squares regression of the particle recovery as a function of the nanoparticle residence time in the lung. The R_0_ is the extrapolation of this regression to a theoretical breath-hold of zero seconds. The measurement was considered reliable if the square of Pearson’s correlation coefficient was at least 0.9 for the regression. A low correlation would indicate significant variations in the execution of the manoeuvre or otherwise suggest a low-quality measurement. No participants were excluded due to unreliable AiDA results. The intrasubject variability of r_AiDA_ has been shown to be less than 3% between healthy subjects with approximately 18 months between measurements.^18^

### Standard lung function tests

Spirometry, IOS and D_LCO_ were performed using the Jaeger MasterScreen–PFT and Jaeger MasterScreen IOS system (Carefusion Technologies, San Diego, California, USA). Spirometry was carried out in accordance with the European Respiratory Society criteria.^27^ FEV_1_ and forced vital capacity (FVC) were measured.

In addition to D_LCO_, alveolar volume (V_A_) and the carbon monoxide transfer coefficient (K_CO_) were collected from the diffusing capacity test. Using IOS, resistance was measured at 5 and 20 Hz (R5 and R20) and reactance at 5 Hz (X5). Spirometry and D_LCO_ were evaluated against Global Lung Function Initiative reference equations.^28^ IOS results were compared against the equations determined by Nowowiejska *et al*.^29^ One participant from the BPD group was unable to perform the diffusion test, and one participant from the control group was unable to complete the IOS test.

### Statistics

Comparisons between groups were evaluated using Kruskal-Wallis one-way analysis of variance on all groups, followed by a Wilcoxon rank-sum test between the individual groups if significance was found. Kurskal-Wallis’ test was chosen due to the heterogeneity in variance between groups as well as the comparatively small number of participants. Associations between variables were investigated by Spearman’s rank correlation. Multiple regression analysis was performed using an ordinary least squares model. The results from the Wilcoxon rank-sum tests were considered significant for p-values below 0.017 after Bonferroni correction for multiple comparisons. Otherwise, values below 0.05 were considered significant. Calculations were performed using MATLAB V.2020a (The MathWorks, Natick, Massachusetts, USA) and Python V.3.10.7 (Python Software Foundation).

## Results

A total of 41 adolescents, 15–17 years of age, were investigated, of whom 25 were born preterm. Of the preterm, 17 had a BPD diagnosis (see table 1). Background variables and lung function are provided in tables1  2 for the three groups: term-born controls, preterm-born adolescents without BPD and preterm-born adolescents with BPD. There were no significant differences between the groups in age, sex, height or weight.

**Table 2:** Lung function including AiDA results

	Term-born controls(n=16)		Preterm without BPD (n=8)		Preterm with BPD (n=17)	
	Mean±SD	95% CI	Mean±SD	95% CI	Mean±SD	95% CI
r_AiDA_ (µm)	231±12	(224, 238)	257±23^*^	(238, 275)	313±54***	(285, 341)
R_0_	0.43±0.13	(0.36, 0.50)	0.48±0.11	(0.39, 0.58)	0.49±0.09	(0.44, 0.53)
r*^2^*	0.97±0.03	(0.96, 0.99)	0.98±0.02	(0.96, 0.99)	0.98±0.02	(0.97, 0.99)
FEV_1_ (z-score)	−0.15±0.71	(−0.53, 0.23)	−0.65±0.68	(−1.22, 0.08)	−1.28±1.37*	(−1.98, 0.57)
FVC (z-score)	0.25±0.71	(−0.13, 0.63)	0.15±0.86	(−0.57, 0.86)	−0.23±1.23	(−0.86, 0.41)
FEV_1_/FVC (z-score)	−0.67±0.76	(−1.08, 0.27)	−1.02±1.49	(−2.26, 0.22)	−1.58±1.21	(−2.2, 0.96)
R5 (z-score)	−0.14^†^±0.21	(−0.26, 0.03)	0.15±0.49	(−0.26, 0.57)	0.04±0.37	(−0.15, 0.23)
R20 (z-score)	−0.01^†^±0.26	(−0.15, 0.14)	0.2±0.36	(−0.11, 0.50)	0.03±0.27	(−0.11, 0.17)
X5 (z-score)	0.15^†^±0.34	(−0.04, 0.33)	−0.46±0.55*	(−0.91, 0.00)	−0.31±0.74	(−0.69, 0.07)
D_LCO_ (z-score)	0.09±0.93	(−0.41, 0.58)	−0.57±1	(−1.40, 0.27)	−0.92±1.01†*	(−1.46, 0.39)
K_CO_ (z-score)	−0.16±0.63	(−0.50, 0.17)	−0.82±1	(−1.66, 0.02)	−0.80±0.92†	(−1.29, 0.31)
V_A_ (z-score)	0.26±0.89	(−0.21, 0.73)	0.36±0.87	(−0.36, 1.09)	−0.19±1.2†	(−0.83, 0.45)

Data are presented as means and standard deviation (SD)SD and 95% confidence interval (CI)CI.

*p<0.017 compared tocompared with controls; **p**p<0.003 compared tocompared with controls; ***p***p<0.0003 compared tocompared with controls.

†Data missing from one person. Data presented as per cent of predicted (Table S1online supplemental table S1) and absolute values (Table S2online supplemental table S2) can be found in the supplementary material.

BPD, bronchopulmonary dysplasia; D_LCO_, diffusing capacity for carbon monoxide; FEV_1_, forced expiratory volume in 1 s; FVC, forced vital capacity; K_CO_, carbon monoxide transfer coefficient; R5, resistance at 5 Hz; R20, resistance at 20 Hz; rAiDAradius Airspace Dimension AssessmentV_A_, alveolar volumeX5, reactance at 5 Hz

### Airspace Dimension Assessment with aerosol

**Figure 2:**
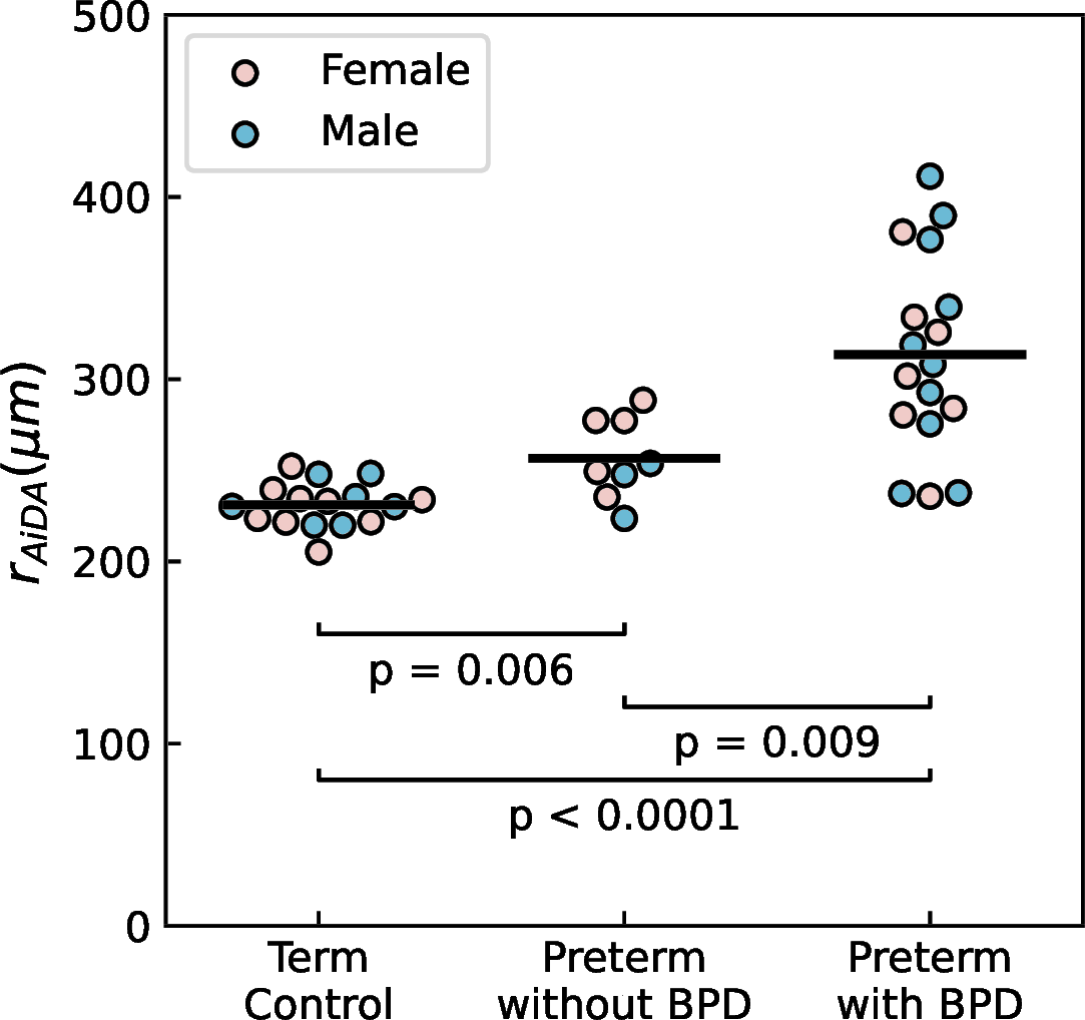
Distal airspace radius (r_AiDA_) for the three groups. The bar represents the median value of each group. BPD, bronchopulmonary dysplasia; r_AiDA_, Distal airspace radius as measured with Airspace Dimension Assessment.

The preterm group had significantly larger distal airspace radii, r_AiDA_, when compared with the term-born controls (295±53 and 231±12 µm, respectively, p<0.0001) and the increase was particularly pronounced in the subgroup of adolescents with BPD versus those without BPD (table 2 and figure 2). There were no significant differences between the groups for the AiDA R*_0_* value. We see no difference in Pearson’s correlation coefficient (r^2^) between the groups, that is, there was no significant difference in the quality of measurement between the groups.

**Figure 3:**
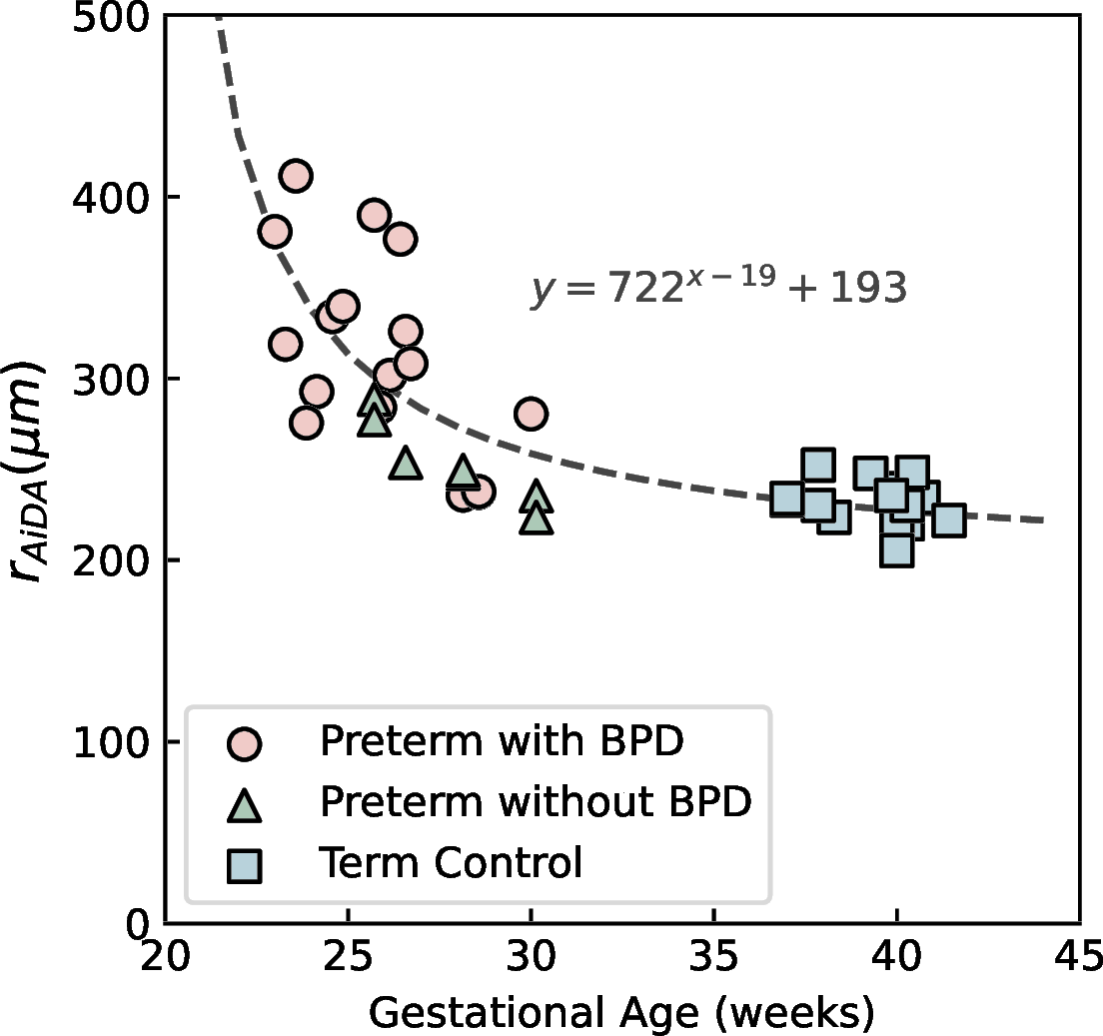
Distal airspace radius (r_AiDA_) as a function of gestational age for preterm participants with BPD (circles), preterm participants without BPD (triangles) and term-born controls (squares). BPD, bronchopulmonary dysplasia; r_AiDA_, Distal airspace radius as measured with Airspace Dimension Assessment.

In the preterm group, r_AiDA_ decreased with increasing gestational age (figure 3) as well as birth weight. The strongest correlation was found between r_AiDA_ and gestational age in the entire study population including both preterm and term adolescents (Spearman’s ρ=0.83, p<0.0001). When investigating the preterm group alone, r_AiDA_ decreased with gestational age (ρ=−0.72, p<0.0001) as well as with birth weight (ρ=−0.70, p=0.0001). We found no relation between gestational age or birth weight and the AiDA results in the control group.

### Standard lung function

FEV_1_ was significantly lower for the BPD group than for the term-born group (p=0.012), while the preterm group without BPD was not significantly different from the term-born group (table 2). There was no significant difference in FVC or FEV_1_/FVC between the groups.

Impulse oscillometry did not demonstrate significant differences in R5 or R20 between the groups. X5 was significantly higher in the preterm group without BPD compared with the term-born group (p=0.005), while there was no significant difference between the BPD group and the term-born group (table 2).

D_LCO_ was significantly lower in the BPD group compared with the term-born control group (p=0.013). There were no significant differences in V_A_ or K_CO_ between the groups (table 2).

The distal airspace radius, r_AiDA_, correlated with FEV_1_ (Spearman’s ρ=−0.52, p=0.0005), FEV_1_/FVC (ρ=−0.43, p=0.005), X5 (ρ=0.32, p=0.045), D_LCO_ (ρ=−0.57, p=0.0001) and K_CO_ (ρ=−0.54, p=0.0003). Other lung function metrics were not significantly correlated with r_AiDA._ The AiDA parameter R_0_ correlated with FEV_1_/FVC (ρ=−0.40, p=0.011) and K_CO_ (ρ=−0.50, p=0.001).

Spirometry, IOS and D_LCO_ data presented as per cent of predicted (online supplemental table S1) and absolute values (online supplemental table S2) can be found in the supplementary material.

### Anthropometric and neonatal data

We found no significant correlations between r_AiDA_ and height, weight, sex or age (table 1). When investigating only the preterm participants, we found correlations between r_AiDA_ and number of days spent in mechanical ventilation (ρ=0.57, p=0.003) and with CPAP (ρ=0.91, p<0.0001) during infancy.

### Multiple regression analysis

A multiple regression analysis was performed to separate the effect of a BPD diagnosis and gestational age on r_AiDA_. For the entire study group, this resulted in a highly significant estimate (*F*(2.37)=25.4, p<0.0001, R^2^=0.578). Both gestational age and BPD diagnosis added significantly to the model (gestational age: β=−0.5, 95% CI: −0.84, −0.15, p=0.007; BPD: β=40.7, 95% CI: 8.4, 72.9, p=0.015). For the prematurely born group alone, it showed that gestational age (regression coefficient, β, of −1.2, 95% CI: −2.1, −0.35, p=0.008) was significantly associated with r_AiDA_, while a BPD diagnosis (β=35.0, 95% CI: −5.6, –75.5, p=0.088) was not. There is some collinearity between gestational age and BPD diagnosis for the preterm group (Spearman’s ρ=−0.36, p=0.07), although not sufficient to exclude one of the variables from the multiple regression analysis.

## Discussion

The prematurely born adolescents had enlarged distal airspaces compared with age-matched controls as measured by AiDA. The difference was most pronounced between the control group and the BPD group and correlated inversely with gestational age.

Some studies indicate that the enlargement of the airspaces seen in infants with BPD can heal during childhood as alveolarisation continues,^30^ while others have found that they remain enlarged even during adulthood.^31^ Our findings support the notion that the alveoli remain enlarged in the preterm group even after bulk alveolarisation has ended after 12–24 months,^32^ and that an earlier arrest leads to larger distal airspaces later in life. Since lung volumes were approximately similar for the preterm group and controls, we assume that the increased alveolar size is an effect of the preterm group having fewer alveoli overall. This agrees with previous findings, where most subjects with BPD showed pulmonary abnormalities during later childhood and adulthood.^31 33 34^

Structural changes in the lungs of preterm born are common finds in studies using CT and results are varied.^1535^,^37^ The results from these studies are not always in agreement, and while most agree that there are pulmonary abnormalities in those born prematurely, the associations between the CT scores and the damage to the lung are not always consistent. Studies by Wong *et al* and Ronkainen *et al*, investigating prematurely born adults from the pre-surfactant era and post-surfactant era schoolchildren, respectively, found that CT results correlated well with FEV_1_ but not with D_LCO_.^16 34^ Similar to our results, these studies both imply that some pulmonary abnormalities remain after infancy in those who were diagnosed with BPD. Hypoattenuation has been shown to correlate with abnormalities in the peripheral lung, which we also observed evidence of in the present study.^15^ Many previous studies identify a need for longitudinal studies on patients who are prematurely born and BPD, in which case CT would not be an ideal technique due to its radiation exposure. AiDA could serve as a practical alternative to conduct such studies.

Both FEV_1_ and D_LCO_ were significantly lower in the BPD group than the control group. The only significant result using IOS was X5, with lower values for the non-BPD preterm group. This unexpected result is likely caused by the gender differences in Nowowiejskas’ reference equations in combination with the variations in the gender makeup between groups in this study. Of the methods used in this study, the AiDA method showed the most striking differences between groups. This indicates that spirometry, IOS and D_LCO_ do not completely capture the extent of the underdevelopment of the distal lung. For D_LCO_ this could be due to a difference in gas exchange due to variations in vascularisation or similar non-structural factors.^38^ It is possible that while the alveoli of the preterm-born are underdeveloped with regards to structure, their ability to absorb oxygen is not affected to the same degree due to compensating factors. For example, preterm birth has previously been shown to be linked to increased haemoglobin levels.^39^ In addition, V_A_ and K_CO_ are not expected to correlate strongly with r_AiDA_, as the degree of alveolar septation could differ significantly between individuals with similar V_A_.

Our research group has previously demonstrated that r_AiDA_ correlates strongly to emphysema detected by CT both visually and defined by densitometry.^20^ In contrast to measures such as spirometry, results from AiDA are almost exclusively dependent on the geometrical structure of the distal lung, which could explain the increased sensitivity. Since the correlation between r_AiDA_ and measurements other than CT are weak it suggests that r_AiDA_ is indeed a measure of emphysema rather than an effect of differences in lung volumes. In a population-based study by Aaltonen *et al*,^17^ the group with CT-assessed signs of emphysema had an average r_AiDA_ of 326 µm, while non-emphysematic participants had an average of 291 µm. In the present study, six participants had an r_AiDA_ above 326 µm, all of whom had BPD. Consequently, we propose that AiDA could effectively identify emphysema in preterm individuals, offering a simpler alternative to CT or MRI imaging and greater precision than spirometry. No correlation has been found between AiDA values and sex or height. Indeed, the only significant demographic variable found is age, with previous studies showing that the average r_AiDA_ increases with age from approximately 240 µm for healthy subjects in their 20s up to around 320 µm for those in their 70s.^25 40^ In this study, the average airspace radius, r_AiDA_, in healthy controls was 231 µm, which is in line with the age-dependent trend seen in previous studies. In these previous studies, we have also observed a trend in the inter-participant variability of r_AiDA_ which increases with age, with the SD being 30 µm in those below 40 years of age and 40 µm for those above.^41^ The SD of 12 µm for the very homogenous control group in the present study is therefore reasonable and supports the previously observed trend. This study is the first to show AiDA results from adolescents, but we expect that the 15–17 years old do not differ from previously studied groups to a degree that would affect the AiDA results further.

In addition to CT, the distal airspace radius measured by AiDA has previously been shown to be strongly correlated with the size of distal airspaces assessed by ^129^Xe diffusion-weighted MRI in a healthy group.^25^ ^129^Xe diffusion-weighted MRI has in turn been shown to correlate with histological results.^42^ In this comparison, we saw no correlation between r_AiDA_ and the heterogeneity index α measured by ^129^Xe diffusion-weighted MRI. We did however see a significant correlation between r_AiDA_ and alveolar size, which suggests that r_AiDA_ is similarly sensitive to heterogeneous ventilation as the alveolar size measured with ^129^Xe diffusion-weighted MRI is. Using ^129^Xe diffusion-weighted MRI, Chan *et al* recently found that children with BPD had a higher apparent diffusion coefficient compared with both prematurely born without BPD and term-born controls.^43^ This is consistent with our results, but the difference between groups is more pronounced in our study which likely is explained by the longer average gestation of the group investigated by Chan and colleagues. Another MRI study, by Flors *et al*, also showed that children with BPD had higher apparent diffusion coefficients than their term-born peers.^31^

AiDA measurements also produce the R_0_ value, which represents the theoretical recovery after zero seconds of particle residence in the lung. This has been suggested to contain some information on heterogeneity of small conducting airways.^19^ In this study, we see no difference in R_0_ between the groups. This is consistent with histological findings by Sobonya *et al*, who found no difference in the size of the small conducting airways in a subject with BPD compared with participants without the diagnosis.^44^ However, the precise physiological factors that govern R_0_ need to be investigated further.

Multiple factors may contribute to pulmonary abnormalities in prematurity as for instance mechanical ventilation, oxygen treatment, gestational age and birth weight. Trying to tease out the importance of each factor is difficult. Even so, our attempt to model this indicates that lower gestational age is associated with a higher r_AiDA_. A cursory investigation on the effects of ventilation as well as surfactant use, indicated that a longer time spent with mechanical ventilation was associated with a higher r_AiDA_ and having received surfactant treatment was associated with a lower r_AiDA_. It is likely that these are important variables to consider, but they could not be reliably investigated in this study due to its small size and the collinearity between variables. We cannot conclude whether a BPD diagnosis itself is a predictor for larger distal airspaces or whether it is mostly an effect of other factors. There was no significant difference in the quality of data across the groups.

Lange *et al* has shown that the accelerated decline in lung function often associated with chronic obstructive pulmonary disease (COPD) is not necessarily present in patients with COPD if they had an initial FEV_1_ lower than normal during early adulthood.^45^ It is likely that subjects with high airspace radius in adolescence will reach r_AiDA_ values typically seen in patients with COPD as they age even without any additional risk factors. Therefore, the adolescents with lung function deficits included in this study may already be on a lower lung function trajectory that will continue throughout life. We suggest that being born extremely pre-term (ie, before week 28) should be seen as a major risk of future pulmonary complications, regardless of a potential BPD diagnosis. Longitudinal measurements of this group are needed to establish any prospects of recovery. Again, AiDA could serve as a practical method to conduct such studies.

This study was conducted on a limited number of demographically homogenous individuals. Thus, extrapolation to the patient–population as a whole is uncertain. We also unfortunately lack neonatal data, most importantly the oxygen content of the ventilation, which could have been used to grade the severity of BPD. We also lack CT or MR imaging, which could have been used to further benchmark the method for this group. It is also difficult to deduce to what degree the increase in airspace radius is due to an arrest in alveolar development or due to treatment during infancy. The increased knowledge on the progression of BPD from this study provides additional information on the long-term consequences of the disease and may be indicative for treatment.

In conclusion, we show that adolescents born preterm have increased size of the distal airspaces compared with term-born controls. This is likely a consequence of the arrest in alveolar development seen during infancy and is dependent on gestational age and the BPD diagnosis. We also found that the distal airspace radius, as measured by AiDA, was the most significant difference between the lungs of prematurely born adolescents and term controls when compared with the results of other lung function tests.

## supplementary material

10.1136/bmjresp-2024-002666 online supplemental table 1

## Data Availability

Data are available upon reasonable request.
