# Supplementary material for: Enlarged airspaces in the distal lung in adolescents born very preterm as measured by aerosol
Source: BMJ Open Respir Res. 2024 Dec 22;11(1):e002666. doi: 10.1136/bmjresp-2024-002666 (PMC11667324; doi:10.1136/bmjresp-2024-002666)
Supplement: online supplemental table 1 [file bmjresp-11-1-s001.pdf]

Supplementary

Table S1: Lung function values presented as percentage of the predicted value.

	**Term born controls (n=16)**	**Preterm without BPD (n=8)**	**Preterm with BPD (n=17)**
FEV_1 (%pred)	98 ± 8	92 ± 8	85 ± 16*
FVC (%pred)	103 ± 8	102 ± 10	97 ± 14
FEV_1 / FVC (%pred)	95 ± 6	91 ± 13	86 ± 11
R5 (%pred)	92 ± 12†	110 ± 30	103 ± 23
R20 (%pred)	102 ± 20†	117 ± 28	104 ± 19
X5 (%pred)	93 ± 21†	127 ± 31*	120 ± 44
D_{LCO} (%pred)	102 ± 13	93 ± 14	88 ± 13*†
K_{CO} (%pred)	98 ± 9	89 ± 14	90 ± 12†
V_A (%pred)	103 ± 10	104 ± 9	98 ± 13†

Data are presented as means and standard deviations. Abbreviations: BPD: Bronchopulmonary Dysplasia; FEV_1, forced expiratory volume in 1 second; FVC, forced vital capacity; R5, Resistance at 5 Hz; R20, Resistance at 20 Hz; X5, Reactance at 5 Hz; D_{LCO}, diffusing capacity for carbon monoxide; K_{CO}, carbon monoxide transfer coefficient; V_A, alveolar volume; * p<0.017 compared to controls; ** p<0.003 compared to controls; *** p<0.0003 compared to controls; †: data missing from one person.

Table S2: Absolute values of lung function.

	**Term born controls (n=16)**	**Preterm without BPD (n=8)**	**Preterm with BPD (n=17)**
FEV_1 (L)	3.64 ± 0.53	3.42 ± 0.57	3.12 ± 0.65
FVC (L)	4.37 ± 0.68	4.29 ± 0.66	4.11 ± 0.68
FEV_1 / FVC (%)	78.3 ± 21.3	80.2 ± 10.9	75.8 ± 10.1
R5 (kPa s L^{-1})	0.33 ± 0.05†	0.39 ± 0.12	0.38 ± 0.08
R20 (kPa s L^{-1})	0.31 ± 0.05†	0.36 ± 0.08	0.31 ± 0.05
X5 (kPa s L^{-1})	-0.09 ± 0.02†	-0.12 ± 0.04	-0.12 ± 0.04*
D_{LCO} (mmol min^{-1} kPa^{-1})	8.61 ± 1.79	7.88 ± 2.29	7.53 ± 1.13†
K_{CO} (mmol min^{-1} kPa^{-1} L^{-1})	1.64 ± 0.14	1.48 ± 0.25	1.54 ± 0.2†
V_A (L)	4.93 ± 0.69	5.3 ± 0.92	4.92 ± 0.79†

Data are presented as means and standard deviation. Abbreviations: BPD: Bronchopulmonary Dysplasia; FEV_1, forced expiratory volume in 1 second; FVC, forced vital capacity; R5, Resistance at 5 Hz; R20, Resistance at 20 Hz; X5, Reactance at 5 Hz; D_{LCO}, diffusing capacity for carbon monoxide; K_{CO}, carbon monoxide transfer coefficient; V_A, alveolar volume; * p<0.017 compared to controls; ** p<0.003 compared to controls; *** p<0.0003 compared to controls; †: data missing from one person.
